# Lateral Humeral Condyle Fracture in Childhood: Results of a New Surgical Technique

**DOI:** 10.3390/jcm13102830

**Published:** 2024-05-11

**Authors:** Giulia Masci, Luca Basiglini, Carlotta Giusti, Angelo Gabriele Aulisa, Francesco Falciglia

**Affiliations:** 1 U.O.C of Orthopedics and Traumatology, Bambino Gesù Children Hospital, IRCSS (Istituto di Ricovero e Cura a Carattere Scientifico), 00165 Rome, Italy; giulia.masci@opbg.net (G.M.); carlotta.giusti@opbg.net (C.G.); agabriele.aulisa@opbg.net (A.G.A.); francesco.falciglia@opbg.net (F.F.); 2 Department of Human Sciences, Society and Health, University of Cassino and Southern Lazio, 03043 Cassino, Italy

**Keywords:** lateral condyle fracture, humerus, Milch and Jackob classification, surgical technique, Baumann angle, carrying angle, children

## Abstract

Fractures of the lateral condyle of the humerus are one of the most common fractures in children, accounting for between 10% and 20% of fractures involving the elbow, with a peak incidence at 6 years of age. Treatment is often surgical for displaced fractures > 2 mm, according to Milch and Jakob classification. There is no consensus in the literature about the appropriate surgical management of these fractures. **Objectives**: The aim of this study is to describe, propose, and evaluate outcomes and complications of the surgical technique of reduction and osteosynthesis using trans-bone suture with resorbable threads. **Methods**: Patients with lateral condyle fractures treated with this surgical technique from 2015 to 2019 were included in this retrospective study, with a minimum follow-up of 24 months. For clinical and functional assessment of the elbow, Mayo Elbow Scores were recorded; we assessed the time of fracture healing, carrying angles, and Baumann angle of the affected limb compared to the healthy contralateral elbow for radiographic data. Complications have also been described. **Results**: We achieved satisfactory results; 36 patients with lateral condyle fractures were included in this study. Radiological healing was achieved in all cases. There was only one complication. No cases required additional surgical procedures. Almost all patients achieved a complete flexion of 110 degrees or more and complete extension. **Conclusions**: This surgical technique has good functional outcomes and fracture healing, a lower incidence of complications when compared to other surgical techniques, and no mechanical failure with good clinical and radiological results.

## 1. Introduction

**Figure 1:**
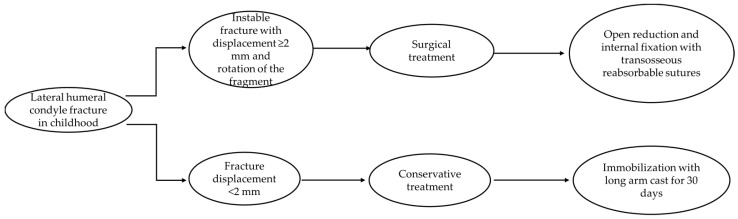
Flow chart for the management of lateral humeral condyle fracture.

Fractures of the lateral condyle of the humerus are one of the most common fractures in children, accounting for between 10% and 20% of fractures involving the elbow [1,2,3]. The average age ranges from 4 to 10 years, with the highest incidence at 6 years of age. The traumatic mechanism is a fall with the hand and elbow extended, a direct trauma to the elbow, or an impact to the valgus that fractures the lateral condyle [4,5,6]. Most of these fractures are isolated, but can often be associated with elbow dislocation and fracture of the radial head and olecranon. Physical examination includes pain, edema, and swelling in the lateral region of the elbow, preternatural deformities, functional impotence, and possible, but rare, neurovascular injury. Undisplaced fractures may not be associated with swelling and deformity and may remain undetected. Radiographic diagnosis is obtained with anteroposterior, lateral, and internal oblique views. Sometimes, it may be necessary to acquire CT images, which have been shown to be very accurate in both diagnosing a fracture and in determining the displacement of the fragment. MRI and ultrasonography can be used for minimally displaced fractures to assess the integrity of the cartilage surface [4]. Milch’s and Jakob’s classifications are the most widely used. Milch’s classification is based on the location and course of the fracture line in relation to the capitellotrochlear groove of the distal humerus; Jakob’s classification is based on the degree of displacement and rotation of the fragment. Milch type I fractures are stable; the fracture line involves the ossification nucleus of the lateral condyle and passes laterally to the trochlear groove, and the trochlear ridge remains intact. Type II fractures, on the other hand, are unstable because there is capsular ligamentous injury, and the fracture line involves the growth nucleus of the trochlea and passes medially to the trochlear groove. This classification is supplemented with the Jakob classification, based on the degree of displacement and rotation of the lateral condyle fragment. It consists of three stages: in stage I, the displacement is less than 2 mm; in stage II, the displacement is between 2 and 4 mm but without rotation of the fragment, with congruence of the articular surface; and in stage III, the displacement is greater than 4 mm, the fragment is rotated, and there is a complete loss of articular congruence [5,6]. Many complications can occur, independent of the type of treatment used. As regards clinical complications, ROM reduction of the affected elbow and the persistence of pain, axial deformities (cubitus valgus or cubitus varus), overgrowth of the lateral condyle with consequent lateral prominence and pseudovarus deformity, and, of course, postoperative infections may be observed. Radiographically, on the other hand, we can observe nonunion and pseudoarthrosis, alterations in the Baumann angle and the carrying angle, avascular necrosis of the ossification nucleus of the trochlea resulting in a fishtail deformity, and premature closure of the growth cartilages. Treatment of these complications depends on the age of the patient, the residual growth potential, and the functional limitation, and often requires further surgery, such as epiphysiodesis, osteotomy, and correction by resection and interposition [4,6,7,8]. Conservative treatment with a brachio-metacarpal cast is recommended in cases of non-displaced fracture, considered stable, to avoid unnecessarily aggressive treatments and the most common related complications. In order to avoid these kinds of complications, treatment is often surgical for displaced fractures of >2 mm, according to Milch (fracture line location) [9] and Jakob (stages of displacement) (Figure 1) [10]. There is no consensus in the literature about the appropriate surgical management of these fractures. Traditionally, open reduction and internal fixation are the methods used to ensure anatomic reduction [11], while several fixation devices have been described (multiple smooth pins, lag screws, threaded pins). Open reduction and pin fixation with Kirschner wires is the most utilized surgical technique described in the literature, owing to the possibility of pinning the physis without physeal damage and allowing for easy removal [6,12]; on the other hand, potential complications could be pin infection, delayed union, and malunion or nonunion of the fractures [6,13,14]. Screw fixation has the advantage of a solid construct with compressive properties [3,15], preventing distal fragment loss of reduction [16]; otherwise, implant revision, growth arrest, and the need for hardware removal under general anesthesia could be sequelae of choosing this kind of fixation [13,14]. In our department, we started to perform a new surgical technique from 2004 for unstable and displaced fractures > 2 mm. We thought that it might be useful to perform an open reduction in order to visualize and directly reduce the displaced distal fragment, alongside an internal fixation technique that could be safe and strong and minimize complications and the risk of reintervention. The aim of this study is to describe, propose, and evaluate outcomes (results and complications) of this surgical technique. There are no recent descriptive studies or case reports regarding this surgical technique in the literature. The Traumatology Unit of Bambino Gesù Children Hospital has been using this technique since 2004, considering it safe and effective, because it allows the anatomical reduction of the fracture, minimizing complications, and it does not require a second surgery to remove the fixation devices once healing has been obtained.

## 2. Materials and Methods

**Figure 2:**
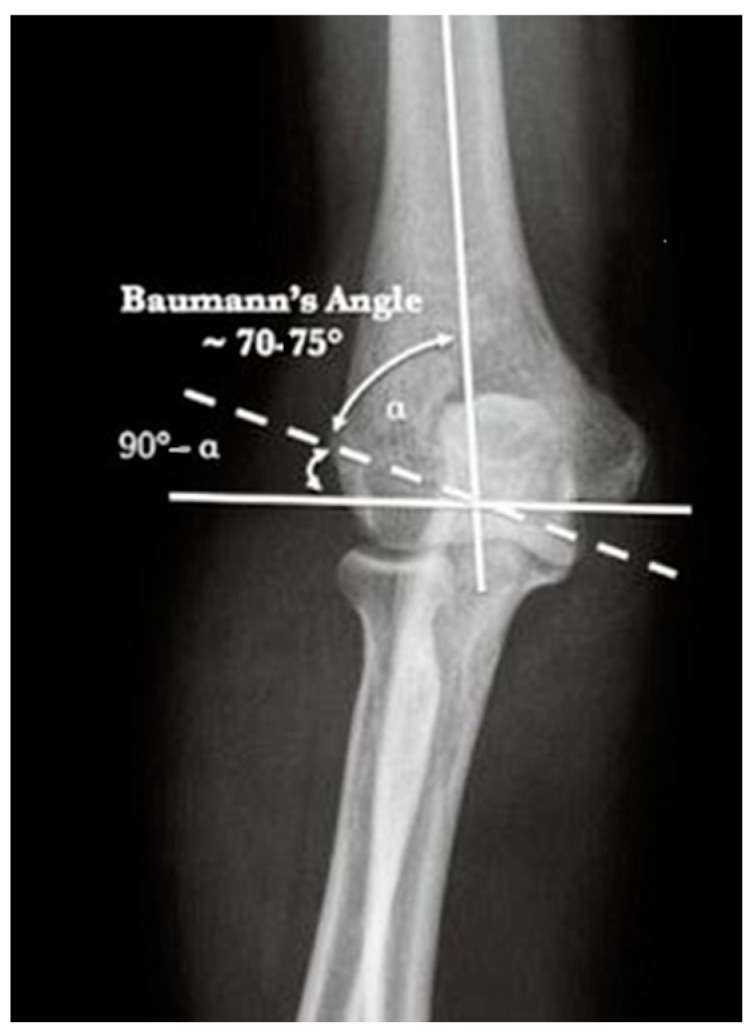
Normal Baumann angle [17].

**Figure 3:**
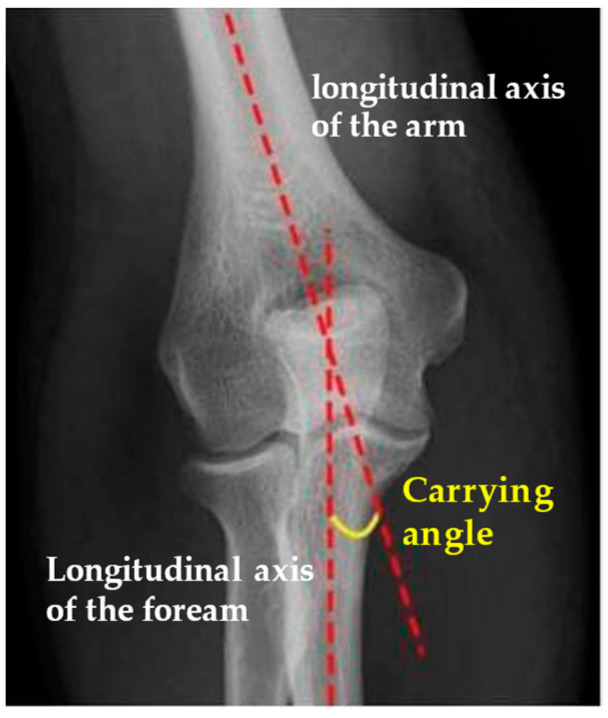
Normal carrying angle [17].

From January 2015 to December 2019, 36 children underwent surgical treatment of a lateral condyle fracture of the elbow in our department; they were all treated with open reduction and internal fixation with transosseous sutures using 0 or 1 Vicryl^®^ OS-4 synthetic absorbable sutures (ETHICON Inc., Johnson & Johnson, Somerville, NJ, USA) depending on the age of the patient. Both clinical and radiographic outcomes were evaluated. Clinical data were assessed by an accurate physical examination. The functional outcome was evaluated according to the Mayo Elbow Score. Range-of-motion recovery, any persistence of pain, and axial deformities were assessed. Radiographic data were processed by re-evaluating the radiographic images performed at follow-up approximately 7 and 30 days after surgery and by a comparative radiographic examination of the elbow in two projections (AP and LL) performed at the time of final follow-up. Baumann’s angle was measured and compared with the healthy side to evaluate the quality of reduction by all authors (Figure 2). In the same way, measurement and comparison of the carrying angle were performed by all authors (Figure 3). The carrying angle of the elbow is determined by the intersection of the longitudinal axis of the arm, and thus of the humerus, and that of the forearm, which is the ulnar axis. Normally, the elbow axis is slightly valgus, but it varies from child to child; there is also variability between boys and girls. Measurements are also not uniform, but once skeletal maturity is reached, they generally tend to reduce and stabilize, decreasing the variability between children. The carrying angle must also be assessed clinically, comparing it with the contralateral one. The Baumann angle is used to assess the alignment of the fracture after the reduction; it is determined by the intersection of the line drawn along the longitudinal axis of the humerus and a line drawn along the conjugation cartilage, between the capitulum humeri and the distal humeral metaphysis. This angle varies from child to child and averages approximately, 72°, and should always be compared with the healthy lateral counter. The Baumann angle and carrying angle are used and assessed by AP projections of the elbow. Exclusion criteria were open fractures, contralateral elbow fractures, and fractures with delayed treatment due to patient complications. Statistical analysis was performed using the GraphPad Prism 5 software (9.4.1.681) The mean values and standard deviations of both clinical data (age, follow-up, VAS score, Mayo Elbow Performance Score, healing time, and surgical time) and radiographic data obtained (Baumann angle and carrying angle) were recorded. Student’s *t* test was developed to analyze the differences between the angles measured in radiographs (Baumann angle and carrying angle) of the affected elbow with those measured in radiographs of the healthy elbow, to test whether there was a statistically significant difference or not. All complications and sequelae of the lesion were evaluated: vicious consolidation, nonunion and fishtail deformity, varus/valgus cubitus, avascular necrosis of the capitellum, epiphysiodesis, growth arrest, ROM reduction, infections, persistence of pain, and trophic alterations of the skin scar.

**Figure 4:**
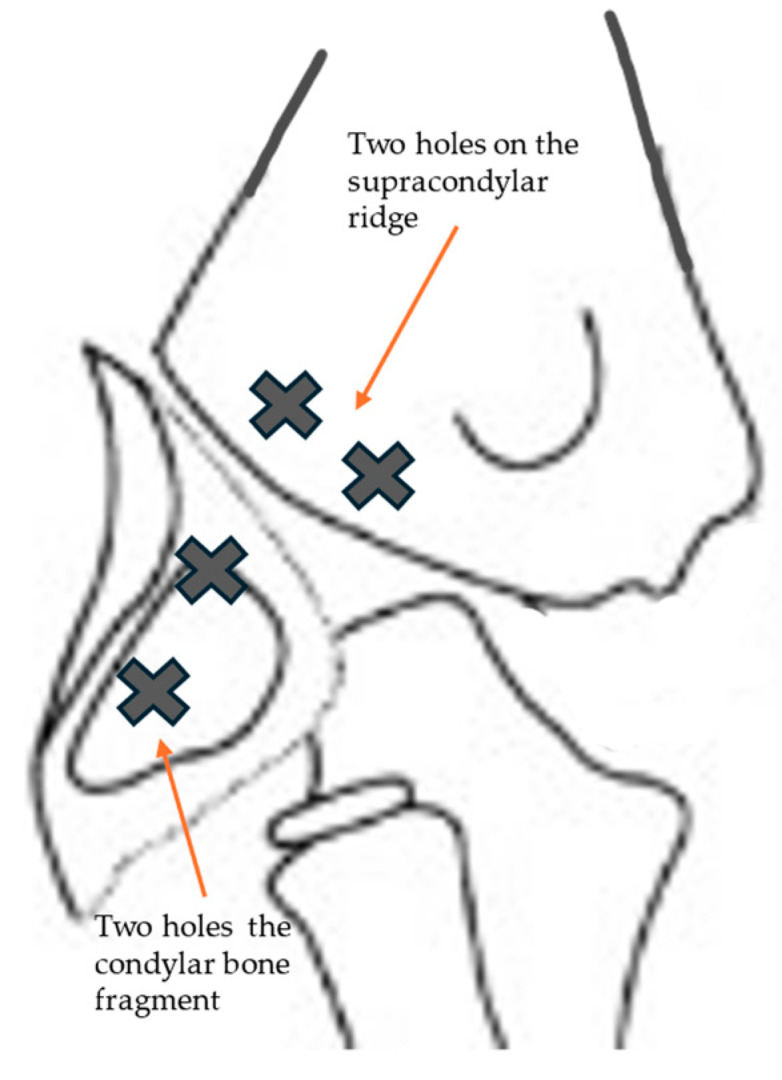
We practiced two transosseous holes with 0 or 1 Vicryl^®^ OS-4 curved needles or with a Kirschner wire set on a drill, thrown on both the supracondylar ridge (the proximal portion of the fracture) and the distal fragment (the condylar bone fragment).

**Figure 5:**
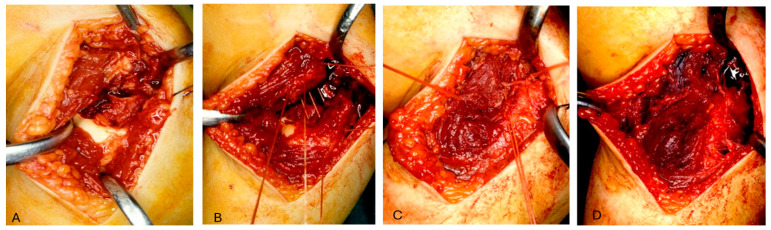
(**A**) The fracture is well visualized after skin incision. (**B**) Two transosseous holes with 0 or Vicryl^®^ needles thrown on both fragments are practiced. (**C**) The bioabsorbable thread is passed inside the holes and a knot is practiced under tension. (**D**) The capsule is closed with a resorbable suture.

**Surgical technique:** The surgery was performed under general anesthesia with supine positioning of the patient with the shoulder in abduction. The skin incision started over and posterior to the supracondylar lateral ridge, proximal to the elbow joint and curved on the lateral surface of the proximal forearm just posterior to the radial capitellum. It should be careful of the radial nerve, running close to the radial head and neck. The muscular fascia was incised over the supracondylar ridge in line with the skin incision. The subfascial exposure should respect the muscle attachments and vascularity, which enter the fragment from behind. The joint capsule was then incised anteriorly over the capitellum and curved over the lateral epicondyle; then, the hematoma and clots were evacuated. Once the fracture was well visualized and reduced, we practiced two transosseous holes with 0 or 1 Vicryl^®^ OS-4 curved needles or with a Kirschner wire set on a drill, thrown on both the supracondylar ridge (the proximal portion of the fracture) and the distal fragment (the condylar bone fragment). The bioabsorbable thread was then passed inside the holes and a knot was practiced under tension (Figure 4). The procedure was repeated a second time to better stabilize the fracture. With the aid of image intensifiers, a 2-projection fluoroscopy image was taken with the aim of documenting the intraoperative reduction and fixation. The capsule was closed with a resorbable 3/0 suture (Figure 5), followed by the muscles and fascia with a resorbable 2/0 suture. Subcutaneous tissue and skin were closed with a fine continuous resorbable suture in order to avoid the stress of nonabsorbable suture removal. A sterile dressing was then applied, and the elbow was immobilized in a traditional long arm cast with the forearm in supination. On discharge, the parents are instructed on the correct home care of the patient and the cast and are referred for clinical and radiographic follow-up approximately seven days later. Removal of the cast is scheduled for approximately 30 to 35 days after surgery, followed by a clinical and radiographic follow-up without cast immobilization; the patient and parents are instructed on active and passive elbow mobilization exercises to restore joint function and muscle trophism. After about a month, the patient is clinically re-evaluated to check that the joint is recovering properly; in cases where the patient is unable to do this independently, cycles of physio kinesiotherapy are recommended.

## 3. Results

**Figure 6:**
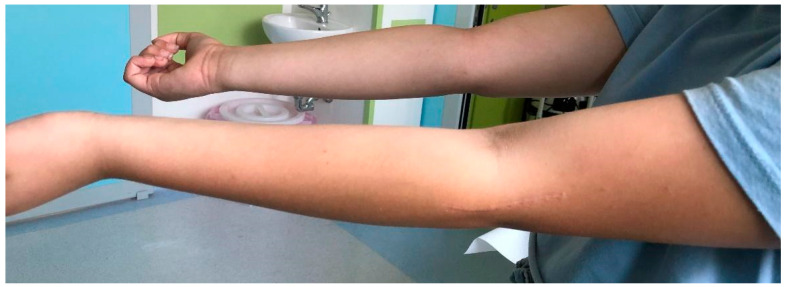
Complete recovery of ROM in extension at 54 months after surgery.

**Figure 7:**
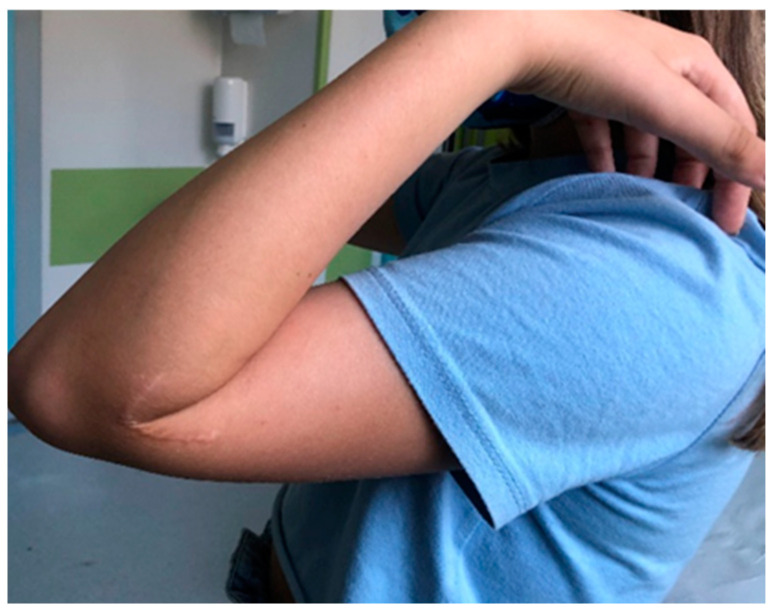
Complete recovery of ROM in flexion.

**Figure 8:**
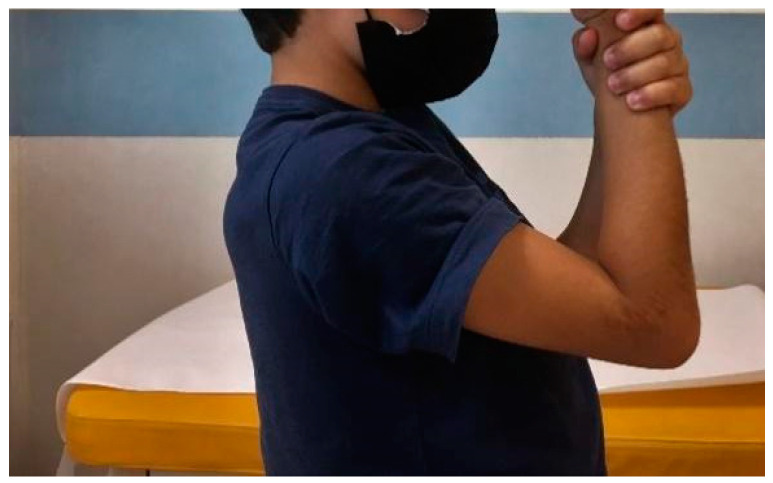
Limitation of flexion at 44 months after surgery.

**Figure 9:**
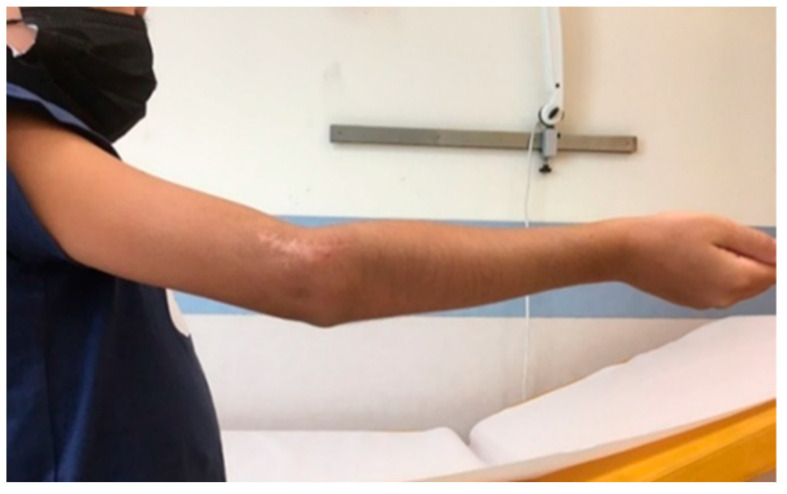
Limitation of extension 44 months after surgery.

**Figure 10:**
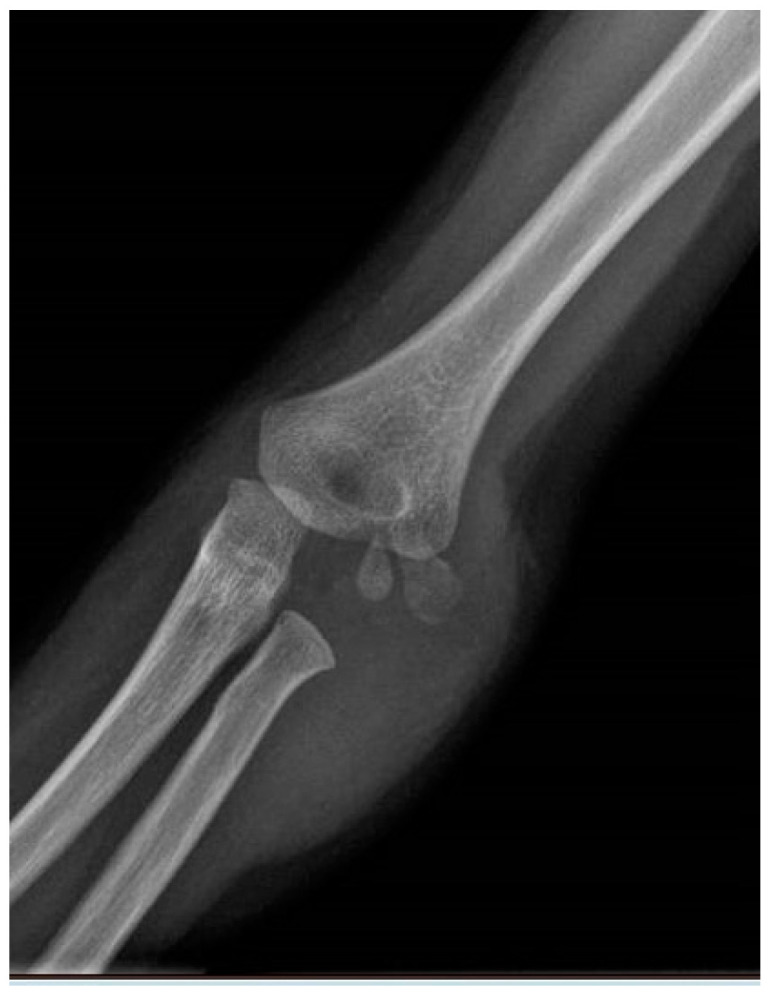
Pre-operative X-ray (anteroposterior view).

**Figure 11:**
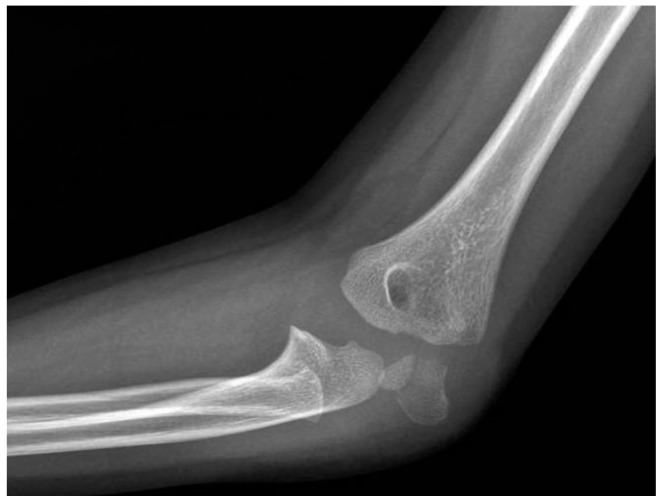
Pre-operative X-ray (lateral view).

**Figure 12:**
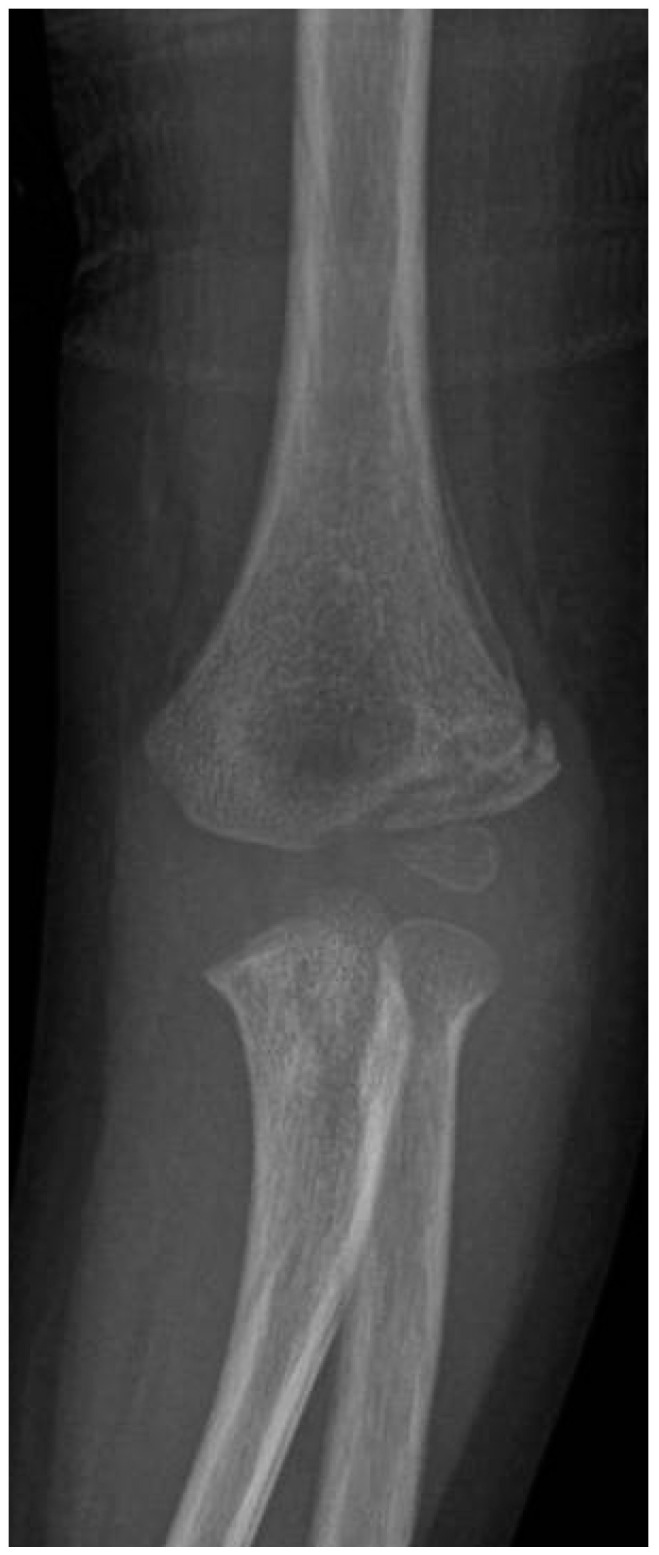
Postoperative X-ray after 30 days of immobilization with long arm cast (anteroposterior view).

**Figure 13:**
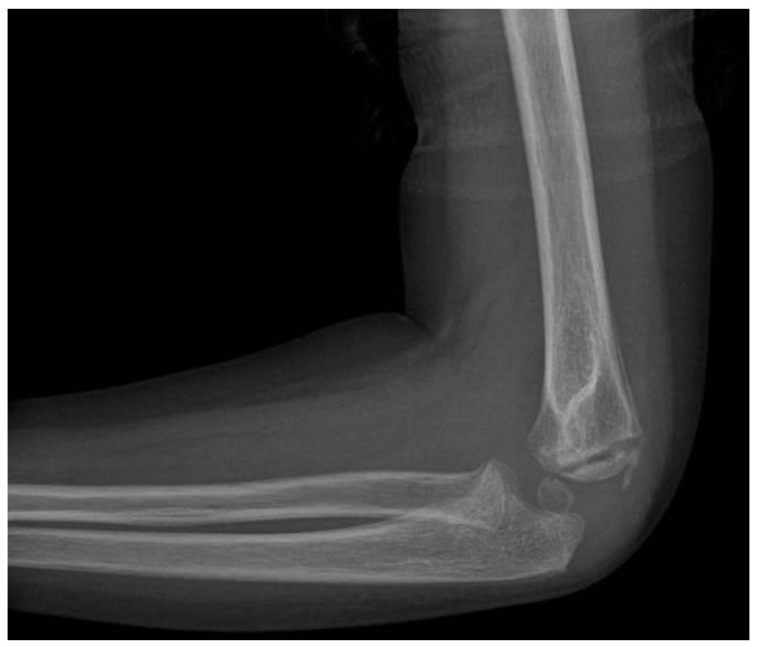
Postoperative X-ray after 30 days of immobilization (lateral view).

**Figure 14:**
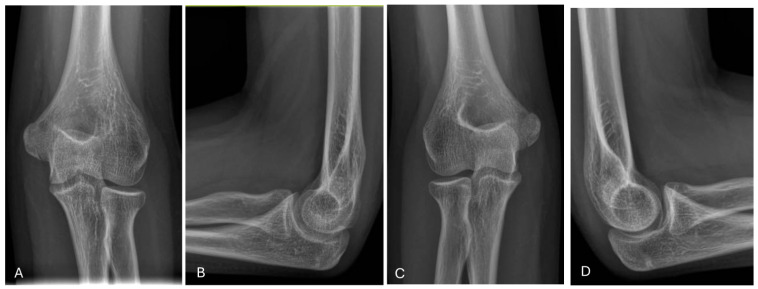
Radiographic follow-up after 73 months after surgery. (**A**) Anteroposterior view of the affected elbow. (**B**) Lateral view of the affected elbow. (**C**) Anteroposterior view of the contralateral elbow (**D**) Lateral view of the contralateral elbow.

From January 2015 to December 2019, a population of 78 patients surgically treated for fracture of the lateral humeral condyle was recorded; of these, 44 required open reduction and underwent surgical reduction and transosseous osteosynthesis with absorbable suture threads. For all of them, surgical treatment was carried out within 72 h from admission in our department. Following the inclusion criteria, one patient was excluded from the study for having suffered a supracondylar fracture of the contralateral humerus one year earlier; the parents of three patients refused to have a further radiographic check-up and X-ray of the contralateral elbow. In addition, it was not possible to contact four patients. The number of patients analyzed in this retrospective study, therefore, was only 36. The average time of follow-up was 42 months, with a minimum of 17 months and a maximum of 73 months. The mean age of the patients evaluated, 21 males (41.7%) and 15 females (58.3%), was 6 years (with a minimum of 2 and a maximum of 11 years) (Table 1). It was necessary to perform a pre-operative CT scan in three cases in order to determine a more accurate degree of fracture displacement. Regarding fracture classification, of the 36 cases, all were classified as Milch type II; 21 were classified as Jakob type III and 15 as type II. At clinical and radiographic follow-up performed in plaster approximately 7 days after surgery, the alignment of the fracture fragments was maintained in all cases and the quality of the reduction was always considered acceptable. The cast was removed after an average of 33 days, and upon removal, on radiographic control without plaster, in all cases, good bone healing was assessed, so all patients and parents were instructed and invited to start functional recovery. In no case was it necessary to reapply the plaster immobilization. At the clinical follow-up carried out approximately 30 days after the removal of the cast, 30 patients had completely recovered the joint ROM, both in flexion–extension and in prone supination; furthermore, in no case was there surgical wound dehiscence. The clinical data collected at the time of follow-up were excellent; in all cases, the Mayo Elbow Performance Score was 100, except one, which was 85. Two patients presented a hypertrophic scar, and a bony subcutaneous callus was found in three patients. No significant axial deviations of the affected elbow compared to the healthy ones were documented (Figure 6 and Figure 7). Only one patient reported a limitation in ROM in flexion–extension (100°–20°), while still maintaining complete pronation–supination (Figure 8 and Figure 9). The radiographic data observed were also excellent (Figure 10, Figure 11, Figure 12, Figure 13 and Figure 14); the calculated Baumann angle was, on average, 78.9 ± 13; the carrying angle was, on average, 12.3 ± 4 (Table 2). The difference between the mean Baumann angles of the affected elbow and the healthy elbow was not statistically significant (*p* = 0.6), neither was the difference between the mean carrying angle of the affected elbow and the healthy elbow (*p* = 0.7). However, a correlation has been identified between the degree of Jakob’s classification with the difference between the carrying angle of the healthy elbow and that of the affected elbow. As regards the surgical procedure, performed by four different operators, the operating time was calculated, which was, on average, 55 ± 16 min. A statistically significant correlation was found between the surgical time and the difference between the carrying angle of the affected and healthy elbow (*p* = 0.037), demonstrating that the degree of displacement and fragment rotation makes fracture reduction more difficult. No major complications were reported; no patient underwent further surgical treatment; and no infections, delayed union, growth arrest, or fishtail deformity were reported.

## 4. Discussion

Fractures of the lateral humeral condyle represent the most frequent intra-articular fracture of the elbow in children of developmental age and the second most common after supracondylar fractures. Unlike supracondylar fractures, lateral condylar fractures rarely result in neurovascular injury [1,2,3]. The peculiarity of this type of fracture is that it is both an intra-articular fracture and a lesion of the growth plate; this means that the treatment must guarantee an anatomical reduction of the fracture and of the joint surface, maintaining the correct rotation of the fragments [18]. In the literature, there is a general consensus that fractures of the humeral lateral condyle that present displacements greater than 2 mm require surgical treatment [6,14,19]. Conservative treatment is recommended in cases of non-displaced fracture, considered stable, to avoid unnecessarily aggressive treatments and the most common related complications such as malunions, pseudarthrosis, avascular necrosis, and growth disorders [6,10]. Non-surgical treatment consists of immobilization in a long arm cast, applied with the elbow flexed to 90°, the wrist extended, and the forearm in neutral rotation. The radiographic control (AP, lateral, and, if it is necessary, internal oblique view) is repeated approximately 4–7 days after the trauma, once the soft tissue swelling has reduced. If no further fracture displacement is documented, the cast is maintained for approximately 4–6 weeks, depending on the formation of the bone callus. Radiographic follow-up is highly recommended because up to 14.9% of fractures will further displace despite the immobilization with a long arm cast. Once the cast is removed, patients and parents are instructed to move the elbow to recover the ROM and joint function, with slow progression to full activity [4]. As regards surgical treatment, to date, analyzing the results of published studies, the superiority of one fixation technique over others has not yet been demonstrated in terms of outcomes and complications [18]. Among the various surgical techniques proposed, closed reduction and percutaneous stabilization with Kirschner wires is recommended when this succeeds in providing anatomical reduction of the fracture. Song et al. have demonstrated that even some displaced fractures can be satisfactorily treated by closed reduction [20]. The fixation technique with metal wires is the most common because it allows enables the stabilization of the fracture without damaging the growth plate [6,21,22]. The metal wires can usually be left exposed to avoid further surgery for their removal, as it has been shown that the infectious risk is not increased [23,24]. When, after an adequate number of attempts at closed reduction of the fracture, the results obtained are not acceptable, we move on to open reduction to effect anatomical restoration of both the fracture heads and the articular surface. Pennock et al., in one study, demonstrated that there was no statistically significant difference in terms of outcome and complications between lesions stabilized with Kirschner wires in a nonoperative manner and those in a surgical manner [25]. Internal fixation can also be performed with cannulated screws, providing greater stability of the construct compared to the placement of two divergent Kirschner wires [3].

Gilbert et al. conducted a retrospective study on 84 patients (average follow-up of 6 months) to compare fixation with Kirschner wires with that with cannulated screws, hypothesizing that the latter guarantees better stability of the fracture and early mobilization of the elbow, minimizing the risks of reduction loss and infectious risk. The screw was positioned in the non-articular portion of the humeral condyle up to its metaphyseal segment. The results obtained demonstrated that fractures treated with screws were associated with a lower incidence of pseudarthrosis and a greater healing speed and early mobilization of the limb, yet requiring a second surgical operation for the removal of the fixation device [13]. In a previous study, however, no significant differences in clinical results and delays in consolidation were found between the two groups examined. However, the group subjected to stabilization with Kirschner wires reported a greater number of infections, functional limitations, and alterations in the carrying angle compared to the other group [22]. The study conducted by Stein also reports good results regarding fixation with Kirschner wires and cannulated screws, yet reporting complications such as functional limitations in extension, deep infections, and growth arrest [14]. Resorbable materials have also been proposed to avoid the second surgical removal procedure, and the studies in the literature describe results comparable with metallic synthesis media [6,26]. A retrospective study conducted by Su et al. compared two groups of patients undergoing reduction and stabilization with Kirschner wires and absorbable screws, for a total of 86 cases, stating that there is no statistically significant difference in clinical and radiographic outcomes between the two groups [27]. Results concerning the application of ultrasound guidance for closed fracture reduction have recently been published in the literature [28]. This technique is very effective because it involves minimal blood loss, minimal surgical incision and no exposure to X-rays, but it is dependent on the skills of the surgeon and cannot be applied to all types of fractures, such as those requiring open reduction.

The studies reported above and analyzed in our study refer to both closed and open reductions. However, there are no studies in the literature regarding the surgical technique of open reduction and synthesis with transosseous sutures. Our study presented epidemiological and radiographic data in line with those published previously [1,24]. Even though it analyzes only one surgical technique, it has as its strength the long follow-up period, allowing the monitoring of possible complications even in the long term. The number of cases analyzed was limited by the fact that the study itself involved the execution of some X-ray control of the traumatized elbow after the injury event and of the contralateral elbow: for this reason, we limited the sample, and some parents, informed as all the others, did not allow the radiographic check to be carried out. However, taking an X-ray of the contralateral elbow was essential to analyze the radiographic outcomes after the operation, as the Baumann angle and the carrying angle present a wide variability among developmental subjects. Analyzing the only case that reported limitation of the ROM, yet without compromising daily and recreational sports activities, it can be observed that the fracture, also visible with 3D CT reconstruction, presented a displacement of more than 4 mm in the pre-operative phase with complete overturn of the fragment, and therefore also required a longer surgical time than the average obtained. The statistical data obtained have in fact demonstrated a correlation both between the difference in the healthy carrying angle with the affected one and the type of fracture according to Jakob’s classification, as well as with the intra-operative surgical time. Four surgeons carried out these operations as first operators, with different ages and experience, but belonging to a homogeneous group. The surgical technique used was the same for each case; the surgical time and treatment outcome were therefore determined by the surgeon’s experience, the displacement of the fracture, and the patient’s age.

Following the pre- and postoperative antibiotic protocol, no cases of infection were recorded; no patient required a second stage of surgery due to a loss of reduction and there were no cases of cast intolerance. Only in two patients, one male and one female, at the clinical check-up two months after the fracture, did hypertrophy of the bone callus occur with consequent pseudovarus of the elbow, which, however, resolved in both cases at the last clinical follow-up. The hypertrophy of the region of the lateral humeral condyle with consequent pseudovarus must be distinguished from the real reduction of the carrying angle and, consequently, from the true varus; both can be a consequence of fractures of the distal end of the humerus at developmental age, as described in the literature, but they have different causes, meaning, and esthetic and functional consequences [29].

## 5. Conclusions

Open reduction and internal fixation with transosseous reabsorbable sutures have proven to be a safe and effective technique for the treatment of displaced fractures of the lateral humeral condyle at developmental age. It does not interfere negatively with physiological development and does not damage the growth plate; it does not require a second surgical operation under anesthesia for the removal of the fixation device. The limitations of this study are the small sample of patients and the lack of comparison with other surgical techniques previously used in our institution to compare rates of complications and radiological outcomes. The strengths of this study are, instead, the long follow-up period and the description of an innovative surgical technique; there are no recent descriptive studies or case reports regarding this surgical technique in the literature.

## Figures and Tables

**Table 1:** Mean values of clinical results.

	Mean
Age	6.06
Follow-up (months)	42.00
VAS score	0.17
MAYO Score	99.58
Healing (days)	33.00
Surgery time (min)	55.42
	Nr
Patients	36
Male	21 [58.3%]
Female	15 [41.7%]

**Table 2:** Values of radiological results.

	Mean	SD	Range
Carrying Angle	12.3°	4.28	5–22°
Contralateral Carrying Angle	12.3°	3.87	5.9–20°
Baumann Angle	78.9°	13.5	60.4–112°
Contralateral Baumann Angle	79.1°	12.7	63–118.8°

## Data Availability

Datasets generated and/or analyzed during the current study are available from the corresponding author upon reasonable request.
